# Computational Modeling of Blood Flow Hemodynamics for Biomechanical Investigation of Cardiac Development and Disease

**DOI:** 10.3390/jcdd8020014

**Published:** 2021-01-31

**Authors:** Huseyin Enes Salman, Huseyin Cagatay Yalcin

**Affiliations:** 1 Department of Mechanical Engineering, TOBB University of Economics and Technology, Ankara 06530, Turkey; hsalman@etu.edu.tr; 2 Biomedical Research Center, Qatar University, Doha P.O. Box 2713, Qatar

**Keywords:** mechanobiology, biomechanics, computational fluid dynamics, fluid–structure interaction, chicken embryo, zebrafish embryo, embryonic development, congenital heart defects, human fetal heart, cardiogenesis

## Abstract

The heart is the first functional organ in a developing embryo. Cardiac development continues throughout developmental stages while the heart goes through a serious of drastic morphological changes. Previous animal experiments as well as clinical observations showed that disturbed hemodynamics interfere with the development of the heart and leads to the formation of a variety of defects in heart valves, heart chambers, and blood vessels, suggesting that hemodynamics is a governing factor for cardiogenesis, and disturbed hemodynamics is an important source of congenital heart defects. Therefore, there is an interest to image and quantify the flowing blood through a developing heart. Flow measurement in embryonic fetal heart can be performed using advanced techniques such as magnetic resonance imaging (MRI) or echocardiography. Computational fluid dynamics (CFD) modeling is another approach especially useful when the other imaging modalities are not available and in-depth flow assessment is needed. The approach is based on numerically solving relevant physical equations to approximate the flow hemodynamics and tissue behavior. This approach is becoming widely adapted to simulate cardiac flows during the embryonic development. While there are few studies for human fetal cardiac flows, many groups used zebrafish and chicken embryos as useful models for elucidating normal and diseased cardiogenesis. In this paper, we explain the major steps to generate CFD models for simulating cardiac hemodynamics in vivo and summarize the latest findings on chicken and zebrafish embryos as well as human fetal hearts.

## 1. Introduction

The cardiovascular system is responsible for delivering oxygen and nutrients to the body [1]. Proper development of the cardiac system has critical importance for human health. Many cardiovascular diseases such as atherosclerosis, heart valve calcification, coronary artery disease, and cerebrovascular disease might develop due to various factors including genetics, altered hemodynamics, and unhealthy lifestyle [2]. In addition to these cardiovascular diseases, various cardiac defects, known as congenital heart defects (CHDs), initiate during the embryonic development such as hypoplastic left heart syndrome, tetralogy of fallot, and bicuspid aortic valve formation [3]. CHDs are considered to be related to the genetic factors; however, these defects are also seen in newborns with no prior CHD history in their families [4]. This observation suggests that genetic factors are not the only reason for CHDs [5,6]. In fact, mechanical forces generated by the blood flow also have an important role in the formation of CHDs [7]. Hemodynamics govern the cardiac development during the embryonic stage and disturbed hemodynamics is considered to be an important source of CHDs [7,8,9,10,11].

Hemodynamics in the cardiovascular system have highly complex behavior depending on the turbulent nature of the cyclic blood flow [12]. Abnormal change in the blood flow may influence the biomechanical environment in the heart. This suggests that mechanobiological forces affect endothelial cell function as well as interstitial and smooth muscle cell function responsible for cardiac growth and remodeling [13,14]. One of the most critical parameters regulating the cell growth and remodeling is wall shear stress (WSS) on the cardiac tissues [15]. The amount of WSS is sensed by the endothelial cells, and a disturbance in WSS level may deteriorate the proper growth [16]. In addition to WSS levels, there are other mechanical parameters such as pressure, shear stress oscillations, and turbulent properties that influence cardiac development.

In the case of CHD detection, a regenerative intervention is required to prevent further growth of the defect. However, it is quite challenging to perform a preventive action in the fetal stage due to surgical limitations and challenges in monitoring [17,18]. The treatment of cardiovascular diseases in newborns and adults can be performed using today’s medical imaging techniques and high-risk surgical methodologies. On the other hand, treatment of CHDs is quite challenging during the fetal stages [19,20,21].

To better understand the development and progression of CHDs, disease phenotypes are experimentally generated and studied in animal models, including mouse [22], chicken [23,24,25], and zebrafish embryos [26,27]. For this purpose, cardiac development is monitored in case of a heart defect which is resembling CHD in the human fetal heart. Etiology of most CHDs is still unclear and further investigations on animals would provide insight about the initiation of CHDs, and shed light for future treatment strategies that can be performed before birth [28].

Vertebrate species are commonly used in the heart development studies due to the conserved developmental processes of the cardiac system. Chicken and zebrafish are typical animal models for investigating the developmental phases in diseased and healthy embryos. The advantages of chicken embryos can be listed as easy access for surgery and imaging, closely resembling human cardiogenesis with four-valve four-chamber configuration, long period of embryonic development which enables long term monitoring, and having less ethical concerns [25]. Zebrafish embryos also have some advantages such as enabling easy genetic manipulation [29]. Zebrafish embryos can be cultured in high numbers, and they are transparent during the early embryonic development, providing an easy access for monitoring using microscopy [30].

In animal experimental studies and clinical observations on CHDs, there are several limitations in monitoring the entire heart development [31]. Therefore, such investigations have to focus on some specific regions which are critical in terms of cardiac development. On the other hand, numerical modeling provides a wider field for spatiotemporal investigations [32]. Using numerical modeling tools, it is possible to determine hemodynamic parameters within the entire flow domain during embryonic development. Therefore, in addition to the experimental and observational studies, numerical modeling approaches are quite useful for elucidating the complex blood flow for investigating the biomechanical regulation of normal and diseased cardiogenesis.

Computational fluid dynamics (CFD) modeling is a widely used numerical approach, enabling to solve the physically governing fluid dynamics equations using medical image-based realistic heart geometries [33]. Fluid–structure interaction (FSI) analysis is an advanced numerical modeling approach, where the flow conditions determined in CFD models are coupled with deformable cardiac tissues [34]. In FSI methodology, the governing equations of fluid and solid domains are solved simultaneously. Using FSI models, it is possible to determine the deformations and flow-driven mechanical stresses on cardiac tissues.

With the development of medical imaging techniques, detailed geometric models can be generated for human fetal hearts [35], chicken and zebrafish embryos [36,37,38,39]. Although there are some challenges in imaging the highly dynamic heart tissues, it is possible to generate four dimensional (4D) models, including three dimensions in space and one dimension in time domain [40]. CFD and FSI models of embryonic hearts can capture the cardiac parameters and hemodynamic disturbances, which provide clues on understanding the etiology of CHDs, human cardiogenesis, and mechanobiological factors playing a role in CHD formation [41].

In this paper, we explain the major steps of generating CFD and FSI models to investigate the cardiac hemodynamics in vivo. Using these numerical modeling approaches, disturbed hemodynamics of defected hearts can be compared to the healthy cases. We also summarize the key findings of CFD studies on chicken and zebrafish embryos, as well as human fetuses.

## 2. Numerical Modeling

In this section, we outline the steps to generate a numerical model of an embryonic heart using the CFD modeling approach. The first step is generating a realistic heart geometry using medical images which are determined via echocardiography, computed tomography (CT), or magnetic resonance imaging (MRI). Medical images are usually obtained in DICOM (Digital Imaging and Communications in Medicine) format as a stock of 2D images after scanning the adjusted heart region with pre-determined slice thicknesses [35]. In order to convert these 2D images into a 3D model, commercial segmentation packages such as Mimics (Materialise, Leuven, Belgium), Vesseg (Carniege Mellon University, Pittsburg, PA), and ImFusion Suite (ImFusion GmbH, Munich, Germany) can be used. There are also open-source segmentation packages such as SimVascular and VMTK. These segmentation packages utilize the contrast difference between the body tissues. The 3D geometry of the blood volume is used as the flow domain in CFD models. Protrusions and internal gaps should be avoided in the CFD model. For this purpose, AngioLab and MeshLab software packages can be used for smoothing and optimizing the geometry [33].

### 2.1. Governing Equations in Solid and Fluid Domains

The governing equations in fluid domain are known as Navier–Stokes equations as given in Equations (1) and (2). Equation (1) expresses the momentum conservation in fluid domain, describing the motion of the fluid particles [42]. Equation (2) is the continuity equation which guarantees the conservation of mass in the flow:

(1) $$\rho_{f}\frac{\partial v}{\partial t}+\rho_{f}\left(v-w\right)\cdot \nabla v-\nabla \cdot \tau_{f}=f,$$

(2) ∇·v=0.

In Equations (1) and (2), the velocity vector of fluid particles is defined by v. If the fluid boundaries are not fixed with zero displacement, the entire fluid domain can move with a velocity vector defined by w. The vector w is equal to zero if there is no solid interacting with the fluid domain. When the fluid field interacts with a solid domain, the vector w is non-zero due to the moving fluid boundaries. The body force vector is defined by f. The main body force is the gravitational acceleration; however, it is neglected in many studies due to limited effect on hemodynamic parameters [43]. Fluid mass density is defined by $\rho_{f}$. Time is defined by t. Fluid stress tensor is defined by $\tau_{f}$ as given in Equation (3), where p is pressure, $\delta_{ij}$ is Kronecker delta, μ is dynamic fluid viscosity, and $\varepsilon_{ij}$ is strain rate tensor [43]. Strain rate tensor is defined in terms of velocity vector as provided in Equation (4):

(3) $$\tau_{f}=-p\delta_{ij}+2\mu \varepsilon_{ij},$$

(4) $$\varepsilon_{ij}=\frac{1}{2}\left(\nabla v+\nabla v^{T}\right).$$

Momentum conservation is the governing equation in solid domain as given in Equation (5), where $\tau_{s}$ defines solid stress tensor, $f_{s}$ defines body forces on the solid, $\rho_{s}$ defines the solid mass density, and $a_{s}$ is acceleration vector of the solid particles. Similar to the fluid domain, gravitational acceleration is the main body force and it is neglected in many studies due to indiscernible effect on solid deformations [44]:

(5) $$\nabla \cdot \tau_{s}+f_{s}=\rho_{s}a_{s}.$$

### 2.2. Meshing and Mesh Independence

**Figure 1:**
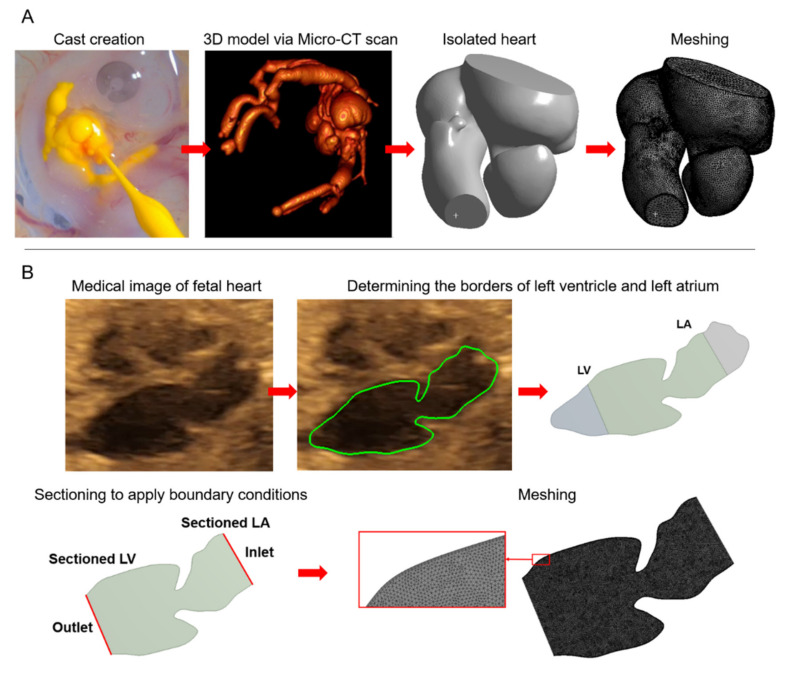
3D and 2D models of computational fluid dynamics (CFD) simulations for a Hamburger–Hamilton 30 stage (HH30) chicken embryo and a 24-week human fetus. (**A**) A cast is created by perfusing Microfil in the embryonic chicken heart. The 3D model is obtained via micro-CT scan. The heart with four chambers is isolated and then sectioned to apply boundary conditions. In the last step, the isolated 3D heart model is meshed to perform CFD analysis. (**B**) Echocardiography image shows four chambers in a fetal human heart. Borders of the left side of the heart are determined and shown by green lines. Left atrium (LA) and left ventricle (LV) are isolated and sectioned to apply boundary conditions. Finally, 2D model geometry is meshed to perform CFD analysis.

Meshing is the spatial discretization of model geometry using finite number of elements. Mesh elements are generally constructed in tetrahedral, hexahedral, or polyhedral form [33,45]. In Figure 1, sample 2D and 3D models are provided for the chicken embryo and human fetus. Previously defined governing equations are solved at each mesh element in order to determine the unknown parameters within the entire flow domain. In CFD analysis, flow velocity, pressure, and WSS are the most important parameters [46]. For the solid domain, mechanical stresses, strains, and deformations have prior interest.

Results of CFD analysis should be mesh independent. In other words, the determined solution of the model should not change with further improvement in meshing. If the solution is changing with the mesh density, it shows that the total element number of the mesh is not sufficient to converge an accurate solution. Here, convergence is generally defined by evaluating the flow rate, pressure, or other flow parameters at a particular region in the flow domain. Usually, the residual of the continuity equation should be on the order of 10^−5^ for a solution to converge [47,48,49]. The total number of mesh elements is required to be increased until reaching solution convergence. If the difference in results is less than 2% for two different mesh densities, the solution can be considered as mesh independent [50]. In the fluid domain, mesh density should be relatively higher at regions close to the wall proximity. In the solid domain, the mesh density should be relatively high in regions with excessive deformations. The effects of cardiac cycles should also be considered in models, as the first three cycles generally lead to computational errors due to transient effects [51].

### 2.3. Material Properties and Flow Characteristics

Selection of appropriate material properties is critically important for accurate CFD analysis. In the fluid domain, blood is generally modeled as a Newtonian fluid, where the dynamic viscosity is assumed to be constant during the analysis. For Newtonian models, blood can be modeled using a mass density of 1.05 g/m^3^ and a dynamic viscosity of 0.035 Poise [52]. In reality, dynamic viscosity of blood changes with the shear rate, which is known as non-Newtonian fluid characteristics [53,54]. For the problems with large flow area, the non-Newtonian effects can be neglected. However, if the flow region is relatively small as in the case of embryonic heart, non-Newtonian effects become important. For non-Newtonian modeling of blood, the Carreau–Yasuda model can be used as given in Equation (6) [10,55,56]:

(6) $$\eta \left(\dot{\gamma }\right)=\eta_{\infty }+\left(\eta_{0}-\eta_{\infty }\right){\left[1+{\left(\lambda \dot{\gamma }\right)}^{a}\right]}^{\left(n-1\right)/a}.$$

In Equation (6), $\dot{\gamma }$ defines the shear rate, η defines the varying viscosity depending on the shear rate, $\eta_{\infty }$ defines the viscosity at high shear rates, $\eta_{0}$ defines the viscosity at low shear rates, and λ, a, n define dimensionless constants. For the blood, these parameters are determined as $\eta_{\infty }$ = 0.00476 Pa s, $\eta_{0}$ = 0.0519 Pa s, a = 0.409, n = 0.191, and λ = 0.438 s [57].

Flow characteristics can be laminar or turbulent in the model. Reynolds number (Re) is a measure to determine the flow behavior, which is defined by the ratio of inertial forces to the viscous forces in the flow. For a pipe flow, Re is found using Equation (7):

(7) Re=ρfVDμ.

In Equation (7), flow velocity is defined by V, and pipe diameter is defined by D. For blood flow investigations, D represents blood vessel or cardiac chamber diameter. The transition from laminar to turbulent flow is observed between Re 2000–2300 [58]. If the Reynolds number is lower than 2000, flow can be modeled as laminar. The vortices in the turbulent flow dissipate energy due to their recirculating motion. For the Reynolds numbers higher than 2000, turbulence models such as K-omega (K denotes kinetic energy and omega denotes energy dissipation rate) and K-epsilon can be employed to obtain accurate flow parameters [46,59,60,61]. These models are known as two-equation eddy-viscosity models due to two extra transport equations representing diffusion and convection of turbulent energy. For in depth analysis, large eddy simulation (LES) or direct numerical simulation (DNS) methods can be used to capture the tiny vortices in the flow [62,63]. In LES and DNS approaches, computational demand is high because the flow mesh is required to be fine enough to capture the smallest turbulent scales in the flow.

In the solid domain, cardiac tissues have hyperelastic and anisotropic material properties [64]. Hyperelastic materials have nonlinear relationship between the stress and strain. The properties of anisotropic materials depend on the direction, which means that the strength of the material is changing with the direction of the applied force. For the models with small tissue deformations, the linearly elastic material model can be employed with a linear relation between the stress and strain. Elastic modulus, Poisson’s ratio, and mass density of the tissue are required to be defined for linearly elastic material model [64]. If there are large deformations in the solid domain as observed in the heart, hyperelastic material models such as the Mooney–Rivlin model should be employed to determine accurate results [65,66,67].

### 2.4. Boundary Conditions

Application of accurate boundary conditions is a critical aspect of numerical modeling. Even with a high-quality geometry and mesh, if erroneous boundary conditions are applied in the model, the results will be misleading. Generally, boundary conditions are defined at inlet and outlet of the flow domain using prescribed pressure and velocity profiles [43]. In the commonly used approach, the inlet is prescribed with a measured flow velocity profile, and outlet is set to a measured pressure [33]. The boundaries other than the inlet and outlet are set as stationary wall boundaries with no-slip condition. The usage of no-slip boundary condition guarantees that the flow velocity is zero on the walls. If there is a moving wall boundary such as in a contracting ventricle, the wall boundaries are set by prescribed motion using dynamic meshes [35]. For FSI models, in addition to the flow domain, solid domain boundary conditions should be implemented. Support regions of solids are generally defined as fixed boundaries with zero displacement and velocity [68]. The regions of solid that are in contact with flow are set as FSI boundaries.

### 2.5. Types of Numerical Models

CFD models can be generated in 2D or 3D, however, 2D modeling is an oversimplification for problems including turbulent flow. Turbulence is inherently a 3D phenomenon, and a 3D model is preferred to obtain reliable results.

CFD simulations are static models unless a prescribed wall motion is introduced, meaning that the vector w given in Equation (1) is zero. CFD analysis can be performed in two ways, namely steady-state and transient analyses. The steady-state analysis gives the final results of the model, and intermediate time steps of the problem cannot be captured. The transient analysis provides the entire solution within the interested time range by employing incremental time steps.

For a more comprehensive investigation, the FSI approach needs to be implemented using the CFD model. In FSI methodology, the flowing blood interacts with the surrounding solid tissues. In order to perform FSI analysis, the wall boundaries in the CFD model should be converted to FSI boundaries for enabling force transfer during blood–tissue interaction. Blood flow generates mechanical forces on the tissues which lead to structural deformations. Solid deformation alters the geometry of the flow model. Therefore, fluid and solid domains counter-interactively change the problem geometry [69]. In FSI analysis, the velocity vector w given in Equation (1) is non-zero, indicating presence of a fluid domain motion due to interactions between solid and fluid.

FSI analysis can be performed using one-way or two-way coupling methods [46]. In one-way coupling, flow-driven forces result in a solid deformation, but this deformation is not reflected to the flow geometry. In other words, flow field is considered as non-deformable in one-way FSI coupling. For the two-way FSI coupling, solid deformation is reflected to the flow domain, and the deformed flow field is regenerated at each computational time step. Therefore, two-way FSI coupling yields more accurate results [42]. Two-way coupling can be employed using either explicit or implicit approaches. If there are high solid deformations in the model, the implicit method should be preferred due to improved convergence [46]. In case of stability problems, relaxation factors can be used or computational time steps of the FSI model can be reduced. 

### 2.6. Parameters for Hemodynamic Assessment

The main parameters for hemodynamic assessment are WSS, time averaged WSS (TAWSS), pressure, flow velocity, and oscillatory shear index (OSI) [70,71]. Pressure is generated on the tissue due to the normal component of flow-driven forces. The tangential component of generated forces results in shear stress. Fluid-driven shear stress on the tissues is known as WSS. Pressure always acts on the normal vector direction of tissue surface. On the other hand, WSS can be effective on any tangential vector, indicating a variability in direction. OSI defines the variability and oscillatory behavior of WSS and it is useful to identify stagnant and circulatory flow zones. The formulations of TAWSS and OSI are given in Equations (8) and (9), respectively [56]:

(8) $$TAWSS=\frac{1}{T}\int_{t-T}^{t}\left|WSS\right|dt,$$

(9) $$OSI=\frac{1}{2}\left(1-\frac{\left|\frac{1}{T}\int_{t-T}^{t}WSSdt\right|}{\frac{1}{T}\int_{t-T}^{t}\left|WSS\right|dt}\right).$$

In Equations (8) and (9), T defines the integration period. If OSI is calculated as zero, it means that WSS is unidirectional. If OSI is equal to 0.5, this expresses that TAWSS is zero.

### 2.7. Uncertainty Quantification and Stochastic Sensitivity Analysis

In most of the cardiovascular CFD studies, flow is simulated for a relatively short time period considering several cardiac cycles. Therefore, the simulations are carried out to resolve the unknown flow variables for a few seconds. This type of analysis is a deterministic approach which does not consider the uncertainties in the flow field. In reality, the inlet boundary waveform is not identical for all cardiac cycles, and there are some uncertainties related to patient-specific characteristics, physical exercise, or resting conditions as observed in computational and experimental flow models [72,73].

In order to capture the long-term possible effects of uncertainties, various scenarios such as different inlet and outlet boundary conditions, turbulent characteristics, and geometric alterations are needed to be investigated using stochastic sensitivity analysis [74]. This way, the influence of uncertainties on the results can be examined for a feasible analysis and the statistical variability of the results can be quantified. There are various techniques for uncertainty quantification in CFD simulations, such as sampling techniques using the Monte Carlo method [75,76], or more compact projection-based methods using polynomial chaos expansions [77,78,79]. As an example, Boccadifuoco et al. (2018) performed a stochastic analysis using the polynomial chaos approach for validation of the CFD findings of thoracic aorta hemodynamics with in vivo measurements [80]. It is stated that stochastic sensitivity analysis is particularly important in biomedical research in case of patient-specific data usage and comparison of experimental and computational results [80].

## 3. Chicken Embryo Models

Chicken embryos are commonly used for elucidating the effect of disturbed hemodynamics on progression of heart defects. The morphologic characteristics of chicken embryo heart development are similar to humans with four chambered heart structures. By performing femtosecond laser photodisruption [24] or micro-surgical operations in the embryonic stage, a variety of cardiac defects can be generated to investigate the hemodynamic parameters before and after the formation of these defects. The most common surgical approaches on chick embryos are vitelline vein ligation (VVL), left atrial ligation (LAL), and outflow tract banding (OTB) [81].

### 3.1. Imaging and 3D Model Generation

3D cardiac geometries of the chicken embryos can be obtained via histology, confocal microscopy, micro-CT, echocardiography, and optical coherence tomography (OCT) [82,83,84,85]. Confocal microscopy provides a high resolution with limited depth of view; therefore, it is preferred for imaging at early embryonic stages [86]. Micro-CT imaging is widely used to obtain 3D geometries and provides the highest resolution [83]. In OCT imaging, back-scattered light is measured up to 1–2 mm depth in cardiac tissue [87,88]. OCT is a non-invasive, non-contact, and high-resolution imaging technique during the embryonic development [89,90,91]. Echocardiography is an ultrasound based imaging method, enabling to image the heart and measure the blood flow rates at specific locations in real time.

Micro-CT cast creation is an invasive method where Microfil (Flow-Tech, Carver, MA, USA) is perfused in the embryonic chicken heart using capillary micro-needles. Microfil solution solidifies into a cast and takes the geometric form of the heart chambers. Then, the embryos are scanned via micro-CT using a voxel resolution around 10 µm [86]. This resolution corresponds to approximately 400 slices on the embryo. After these steps, a 3D model of heart can be generated using segmentation software packages as previously mentioned. Micro-CT can be applied on living animals using clinical agents such as Vivipaque [23]. For imaging living embryos, motion canceling algorithms can be employed to eliminate the geometric errors due to the body movements of the embryo [92].

### 3.2. In Vivo Blood Velocity Measurements

Boundary conditions of CFD models can be determined by measuring blood flow velocities using Doppler ultrasound or Doppler OCT techniques [93,94]. For blood velocity measurements, an aqueous contact zone is prepared between the ultrasound probe and embryo. B-mode echocardiography and Doppler velocity profiles can be determined around the atrioventricular (AV) canal or outflow tract (OFT) of chicken embryos. Using the measured blood flow velocities at the AV canal or OFT, an inlet velocity profile can be calculated for CFD analysis. The inlet velocity profile of the model can be adjusted until CFD-determined velocity and Doppler-measured velocity are consistent [46,95].

### 3.3. CFD Results of Chicken Embryo Models

AV canal, OFT, and aortic arch are the mostly investigated regions in chicken embryo CFD models [38,89,96,97,98,99,100]. Numerical investigations can be categorized as the studies examining normal cardiac development and studies investigating the development after external interventions.

#### 3.3.1. CFD Studies on Normally Developing Embryonic Chicken Hearts

Chicken heart originates as the symmetric mesodermal fields fuse to form a tubular structure [95]. Early embryonic development starts with formation of the primary heart tube at Hamburger–Hamilton stage 8 (HH8). The heart tube starts to loop at HH9 for transforming into a C-shape [101]. Following the looping, the maturation of the primitive heart is initiated until the formation of four heart chambers [102]. Atrial septation starts at HH16, and pre-valve leaflet formation begins at HH21. Epicardium formation and heart septation are completed at HH27 and HH30, respectively [101]. In the literature, CFD studies mostly cover the developmental stages up to HH30 using static or dynamic meshes. The static models employ the same mesh through the cardiac cycle by assuming negligible change in flow geometries. Dynamic models mimic the motion of moving walls; therefore, dynamic models predict more accurate results due to reflecting geometric alterations in the flow model.

In one of the earlier works with static CFD meshes, Yalcin et al. (2011) quantified evolving hemodynamics of AV canal using normal anatomic geometries at the stages of HH17 (52–64 h) and HH30 (6.5–7 days) [95]. At HH17, the flow in the tubular heart is almost laminar with parallel flow streamlines. With the development of cushions and septal walls which are primitive form of valves, recirculating vortices simultaneously appear in the flow domain. WSS levels are investigated around the valve-forming regions, and peak WSS values are determined as 19.34 dynes/cm^2^ at HH17 and 287.18 dynes/cm^2^ at HH30, indicating a severe increase in shear stress with heart development. Spatiotemporally averaged WSS is reported as 3.62 dynes/cm^2^ and 9.11 dynes/cm^2^ at HH17 and HH30, respectively.

In a follow up study, the same group examined WSS in OFT using static models for chicken embryo hearts at developmental stages HH16 and HH30 [32]. Peak WSS values in OFT are reported as 18.16 dynes/cm^2^ at HH16 and 671.24 dynes/cm^2^ at HH30. Spatiotemporal averages of WSS also displayed a monotonic increase from 3.03 dynes/cm^2^ at HH16 to 136.5 dynes/cm^2^ at HH30. Computationally determined flow streamlines, suggesting a lack of mixing in the flow at the early stages of heart development [32]. 

Wang et al. (2009) investigated aortic arch morphogenesis using static models at HH18 and HH24 [103]. It is stated that WSS distributions in the aortic arch shift into a spatially-complex form with the embryonic development as a result of extensive anatomical changes in size and curvature. Tan et al. (2015) also investigated WSS levels using static models for various vasculature types at HH27 [92]. The average WSS levels at peak flow rate are reported as 4.6 dynes/cm^2^ at carotid arteries, 18.9 dynes/cm^2^ at pharyngeal aortic arches, 2.4 dynes/cm^2^ at left and right dorsal aortae, and 7.3 dynes/cm^2^ at common dorsal aorta.

In recent studies, more complicated dynamic mesh approaches are used for such analysis. For this purpose, 2D image sequences are acquired over the cardiac cycle to generate a dynamic mesh. Liu et al. (2012) investigated WSS patterns in OFT using dynamic meshes of HH18 embryos [104]. It is concluded that non-uniform spatial and temporal WSS distributions provide biomechanical cues to cardiac cells which influence the extensive growth and remodeling processes [96,104].

Ho et al. (2017) generated a 4D CFD model with dynamic mesh using normal HH25 embryonic chicken heart and nearby arteries [105]. The dynamics of heart wall motion are reflected to the model using ultrasound-based images. WSS levels are determined within the range of 1.0–5.0 dynes/cm^2^ on the ventricular wall, where the left side of common ventricle experiences lower WSS compared to the right side. It is stated that the net forward flow is optimized in the embryonic heart even in the absence of valves [105]. In Table 1, WSS findings of CFD studies in the literature are presented for various regions in normally developing chicken embryonic hearts considering different developmental stages.

#### 3.3.2. CFD Studies on Cardiac Defects in Chicken Embryos

In order to investigate the influence of hemodynamic disturbances on cardiac development, animals are surgically interfered in many studies. Kowalski et al. (2014) studied left atrial ligation (LAL) on chicken embryo, resembling a CHD known as hypoplastic left heart syndrome (HLHS) [106]. After ligation of the left atrium, chamber volume and blood flow rate are reduced in the left side of the developing heart, showing that LAL immediately alters the hemodynamic behavior. Due to the occlusion in the left side of the heart, the flow is shifted towards the right side, which increases flow streams on the right common cardinal and right vitalline veins. CFD results reveal that WSS is reduced at the left side of the common ventricle and left AV canal depending on the flow reduction due to LAL interference.

Aortic arch is the bended region in the main artery which connects the ascending and descending aorta. Lindsay et al. (2015) investigated interrupted aortic arch morphologies using CFD models [107]. Isolated occlusions in the pharyngeal arch artery are reconstructed using medical image-based embryonic models at HH18 and HH24 [108]. These embryonic stages correspond to 3-days and 4.5-days chicken embryos. CFD results show that pressure gradients and flow redistributions are mostly affected by the occlusion of IVth arches [109]. The highest WSS levels are observed at the narrowest arch diameters due to the decreased flow volume [103].

Outflow tract (OFT) distally connects the heart with the arterial system, and many of CHDs originate around OFT [45,110]. Menon et al. (2015) constricted the ventricle junction/OFT of HH16/17 (51–64 h) chicken embryos using banding intervention [111]. CFD analyses reveal that spatially-averaged WSS on OFT significantly increased due to the banding [112]. Spatially-averaged WSS levels are determined as 0.97 ± 0.26 Pa and 6.1 ± 2.19 Pa on control and constricted OFTs, respectively. Peak WSS is observed as 3.30 ± 0.50 Pa on control heart and 17.8 ± 5.96 Pa on banded hearts, showing a nearly 6-fold increase in WSS levels. In another CFD study on OFT, peak WSS is reported as 18.16 dynes/cm^2^ at HH16 (51–56 h) and 671.24 dynes/cm^2^ at HH30 (6.5–7 days), and spatiotemporally averaged WSS is reported as 3.30 dynes/cm^2^ at HH16 and 136.50 dynes/cm^2^ at HH30 [32], showing an extensive increase in shear levels with the development of the chicken embryo. Liu et al. (2012) reported the effects of OFT on HH18 (3 days) embryo, where the non-uniform spatial and temporal stress distributions in OFT walls provide biomechanical cues on extensive differential growth observed in normal development [104]. The maximum WSS is observed at peak flow rate in the cardiac cycle because of the increased friction between the blood and wall [45].

**Figure 2:**
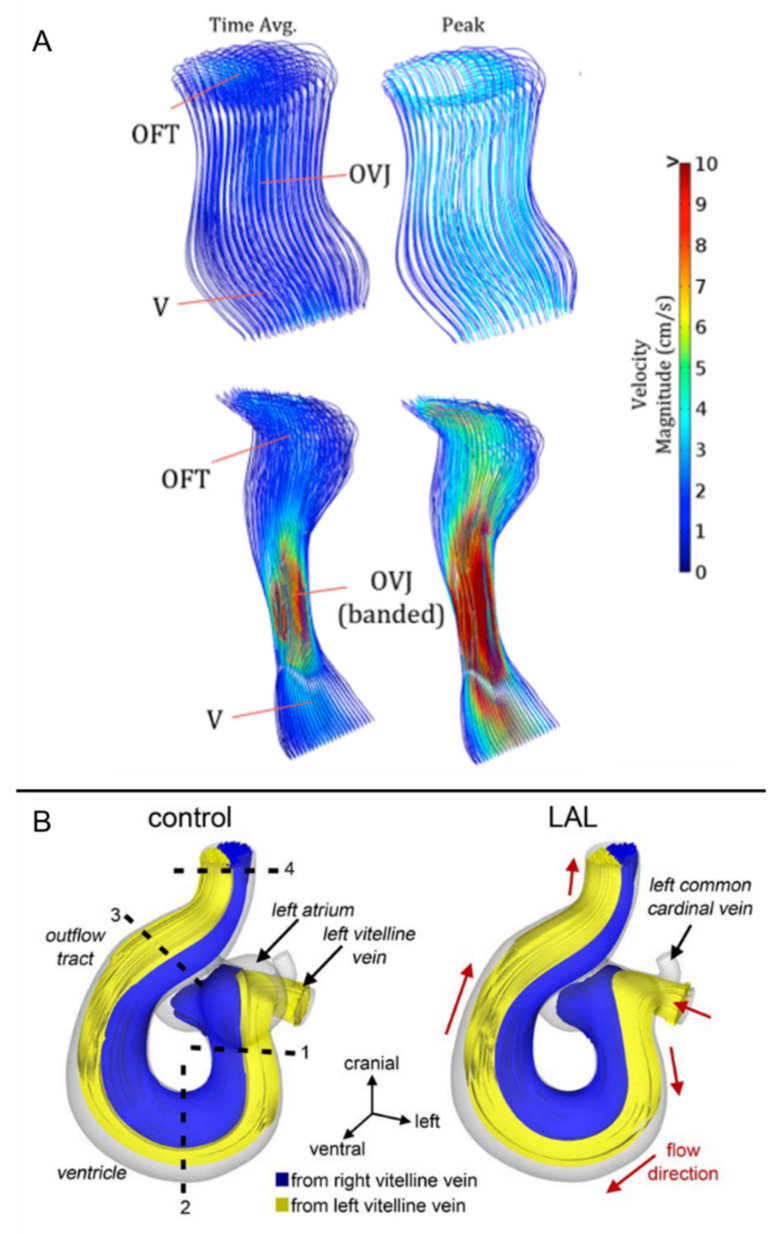
Blood flow velocity streamlines in normal and defected embryonic chicken hearts. (**A**) Peak and time-averaged velocity magnitudes on control (at top) and OFT banded (at bottom) chicken hearts at Hamburger–Hamilton stage 16–17 (HH16–17). V: ventricle, OVJ: OFT/ventricle junction (Reproduced from Menon et al. (2015) [111]). (**B**) Intracardiac flow patterns in normal and left atrial ligated (LAL) embryonic hearts at HH21. Blue and yellow streamlines represent the flows emanating from right vitelline vein (RVV) and left vitelline vein (LVV), respectively (Reproduced from Kowalski et al. (2014) [106]).

**Figure 3:**
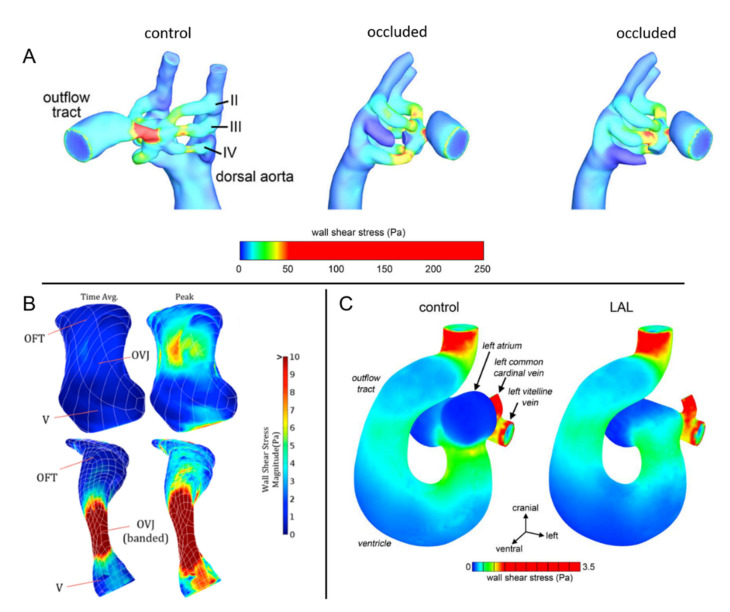
WSS comparison of normal and defected chicken embryos. Significant alterations in WSS are observed between normal and defected embryos. (**A**) Peak WSS on control and occluded chicken embryos at Hamburger–Hamilton stage 18 (HH18). Right lateral aortic arches are shown by II, III, and IV (Reproduced from Lindsey et al. (2015) [107]). (**B**) Peak and time-averaged WSS on control (at top) and OFT banded (at bottom) chicken hearts at HH16-17. V: ventricle, OVJ: OFT/ventricle junction (Reproduced from Menon et al. (2015) [111]). (**C**) WSS distribution on control and left atrial ligated (LAL) embryos at HH21 (Kowalski et al. (2014) [106]).

In Figure 2 and Figure 3, blood velocity streamlines and WSS distributions are provided for normal and distorted embryonic hearts. It is observed that the biomechanical environment and shear stress distributions are significantly different for normal and defected cases.

## 4. Zebrafish Embryo Models

Zebrafish became a widely used animal model to investigate the cardiac defects [113]. Easy genetic manipulation of zebrafish embryos enables to generate cardiac defects resembling human CHDs. Adult zebrafish has two chambered heart with one ventricle and one atrium [30]. Pre-cardiac development starts after five hours post-fertilization (hpf) [114]. Embryonic heart takes the form of a linear tube at 16 hpf [115,116]. The heart movements start around 24 hpf as peristaltic motions. The heart converges into S-shape around 33 hpf, and heart chamber contractions start at 36 hpf. After this stage, primitive valve leaflets begin to develop around 40 hpf for preserving a unidirectional flow [117,118]. After 2 days of post-fertilization (dpf), the ventricular canal takes a curved shape with pronounced internal and external curvatures. Up to 3–4 dpf, the zebrafish embryo is transparent which enables easy access for monitoring embryonic cardiac development [68,119,120].

### 4.1. Microscopic Imaging of Zebrafish Embryo

The amount of blood flow and WSS can be quantified using brightfield microscopy by tracking the red blood cells (RBCs) and ventricle wall movements [119]. Reflective particles can be injected in the flow domain or fluorescently labeled cells can be tracked to determine the flow rate. Blood flow velocities can be obtained in vivo by averaging the velocities of the selected particles. This method is known as digital particle imaging velocimetry (DPIV) [121]. In a cardiac assessment, mean flow velocity at the dorsal aorta is measured as 291 μm/sec at 2 dpf and 766 μm/sec at 6 dpf using the DPIV method [122]. In order to determine the detailed flow hemodynamics of the contracting heart, 4D imaging is required using fast image acquisition (70–85 frame per second) due to the high heart beat rate (2–4 beats/sec) of the zebrafish embryo [123,124,125].

### 4.2. CFD Studies for Normally Developing Zebrafish Embryo Hearts

**Figure 4:**
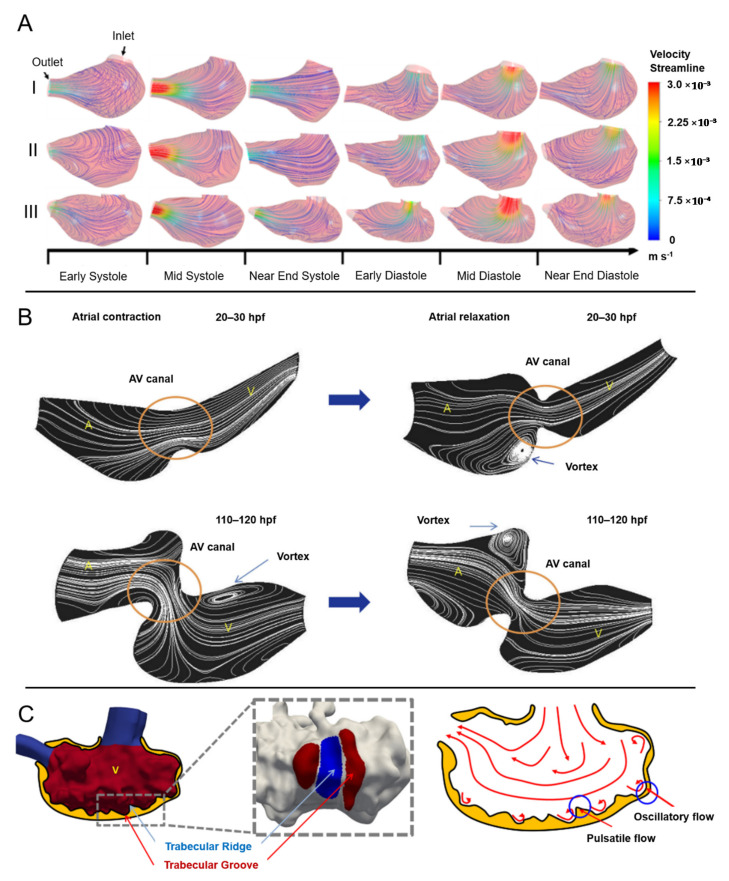
Blood flow in the embryonic zebrafish heart. (**A**) Velocity streamlines for three normal ventricles over the cardiac cycle. During the diastole, laminar flow with high velocity is observed at the ventricular inlet (Reproduced from Yin et al. (2020) [132]). (**B**) Instantaneous flow streamlines at atrial contraction and relaxation for different embryonic development stages ranging from 20 to 120 hpf. The atrioventricular (AV) canal is shown in the circle. At later stages of development, rotating vortices are observed in the ventricle during atrial contraction, and in the atrium during ventricular contraction. A: Atrium, V: Ventricle (Reproduced from Lee et al. (2013) [133]). (**C**) Ventricular trabeculation is observed at later stages (after 3 dpf) of development, resulting in pulsatile flow at ridges and oscillatory flow at grooves (Reproduced from Lee et al. (2018) [15]).

Embryonic cardiac development in zebrafish is influenced by the biomechanical alterations in the flow domain [126,127]. Flow velocity pattern, WSS, and transmural pressure alter the vascular and valvular morphogenesis [128,129,130]. During the contraction phase of the ventricle, a highly dynamic flow field is generated due to the pulsatile nature of the blood flow. With the development of the heart, trabeculation is observed in the ventricle which also increases the complexity of the flow dynamics due to initiation of recirculating vortices inside the trabecular grooves [131]. Flow patterns and velocities determined in CFD studies are provided in Figure 4.

Miller (2011) modeled a 4.5 dpf embryonic heart using 2D CFD simulations [134]. Two different approaches were used for modeling the heart. In the first approach, the atrium and ventricle are considered as rigid structures and a steady flow is prescribed at the inlet boundary. In the second modeling approach, atrium and ventricle are modeled as deformable structures and the flow is generated with the atrium contraction. In both analyses, peak WSS is observed on the endocardial cushions. For the deformable model in the second approach, peak WSS is determined as 70 dynes/cm^2^. This value is in agreement with the findings of Hove et al. (2003) where the peak WSS is stated as 76 dynes/cm^2^ for embryonic hearts at 4.5 dpf [135]. In a recent study performed by Foo et al. (2019), 4D CFD model is employed for 5 dpf zebrafish ventricles [132]. Peak WSS levels are obtained as 37.5 dynes/cm^2^ at inflow tract and 130 dynes/cm^2^ at outflow tract. At mid-ventricular segment, peak WSS is determined within 4–11 dynes/cm^2^ which is quite low compared to inflow and outflow tracts.

In the model of Lee et al. (2013), wall displacements and blood flow rates are measured using DPIV and these measurements are applied as boundary conditions of 2D CFD models [133]. Pre-determined ventricle wall displacements are used as prescribed movements of the dynamic mesh. CFD results are validated by comparing the findings of DPIV measurements. Peak flow rate is observed at AV canal with velocities of 1.0 mm/s at 30 hpf, 3.5 mm/s at 70 hpf, and 5.0 mm/s at 120 hpf. Average WSS levels at AV canal are reported as 3.5 dynes/cm^2^ at 20–30 hpf, 20 dynes/cm^2^ at 40–50 hpf, and 80 dynes/cm^2^ at 110–120 hpf, revealing a significant increase in flow rates and WSS levels as the embryo develops. In another 2D CFD model for 36 hpf and 48 hpf hearts, it is reported that endocardial cells are exposed to oscillatory forces, and the cell localization on the valve-forming region is influenced by the mean WSS direction and oscillatory shear stress gradient [136]. This fact shows that spatial and temporal patterns and variations of WSS are critical in terms of cell growth and remodeling.

Gomez-Garcia et al. (2018) investigated nanoparticle accumulation in 52 hpf zebrafish embryo using a 3D CFD model [39]. For TAWSS higher than 0.033 Pa, an inverse relationship is found between the particle accumulation and TAWSS, indicating that high shear stress avoids particle accumulation on the wall. Interestingly, there is no accumulation when TAWSS is lower than 0.028 Pa. Nanoparticle accumulation is observed within the range of 0.028–0.256 Pa with peak accumulation at 0.033 Pa. In Table 2, WSS levels of CFD simulations in the literature are summarized for normally developing zebrafish embryo hearts considering different developmental stages.

### 4.3. CFD Studies on Defected Embryonic Zebrafish Hearts

**Figure 5:**
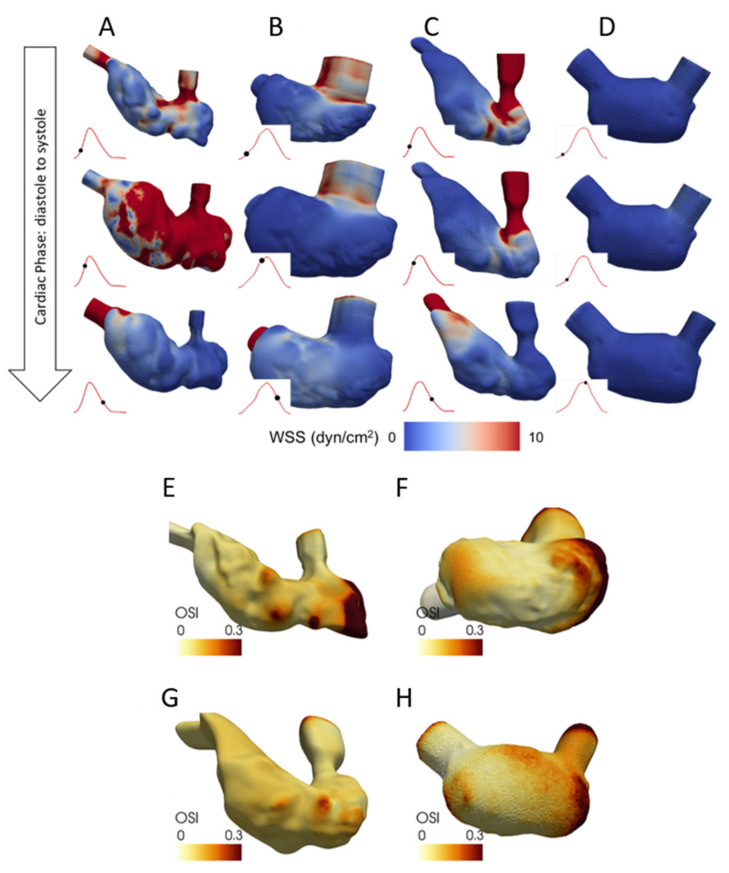
WSS patterns at various instants over the cardiac cycle. (**A**) Normal embryo. (**B**) Inhibited trabeculation. (**C**) Inhibited proliferation. (**D**) Inhibited ventricle development. Red line and black dot represent the instant over the cardiac cycle. OSI patterns are determined using three cardiac cycles. (**E**) OSI pattern of the normal embryo. (**F**) OSI pattern for inhibited trabeculation. (**G**) OSI pattern for inhibited proliferation. (**H**) OSI pattern for inhibited ventricle development (Reproduced from Vedula et al. (2017) [31]).

Vedula et al. (2017) performed 4D CFD analysis using light-sheet imaging of 4 dpf zebrafish embryo [31]. A normal heart is compared to three defected hearts with inhibited trabeculation, inhibited proliferation, and inhibited ventricle development. Moving ventricle walls are used as boundary conditions with pre-defined wall displacements. Generated CFD meshes are composed of 3 to 10 million tetrahedral elements. A total of 4 cardiac cycles were investigated in the analysis using a time step of 0.2 ms. Results of first cardiac cycle were not used in order to prevent transient effects at the beginning of the simulation. Endocardial WSS and OSI patterns of normal and defected embryonic hearts are provided in Figure 5 [31]. A spatially inhomogeneous WSS pattern is observed for the normal heart. On the other hand, defected hearts resulted in homogeneous WSS patterns. In the same manner, the OSI pattern of normal embryo is different from that of disrupted embryos.

It is worthy to note that the main difference in 3D and 4D models is related to the moving boundary walls of the mesh. In 4D models, time dependent wall motions are pre-defined using dynamic mesh. However, 2D and 3D models have fixed walls with stationary mesh.

Lee et al. (2018) generated 4D CFD models of genetically altered zebrafish embryos to demystify the effect of spatial and temporal WSS variations on ventricular trabeculation [15]. Trabecular grooves are exposed to oscillatory flow, on the other hand, a pulsatile flow behavior is observed on trabecular ridges, indicating that a relatively increased pulsatile WSS pattern is exerted on the ridges. Higher levels of OSI, kinetic energy dissipation, and vortex formation are observed in trabeculated ventricles compared to untrabeculated ones [131]. Genetic manipulations are performed on zebrafish embryos to reduce the shear stress and attenuate trabeculation in ventricles. CFD results reveal that trabeculation has a role to preserve ventricular structure and ventricular contraction, and activation of WSS in endothelial cells regulates cardiac trabeculation during the embryonic development [15,123].

## 5. Human Fetal Heart Models

There are a limited number of CFD studies on human fetal heart development due to the challenges in imaging. Fetal intracardiac flow can provide clues for the development of CHDs by assessing the anomalies during the embryonic stage. Here, we summarize the latest CFD studies performed on human fetal hearts.

### 5.1. CFD Studies on Normal Human Fetal Hearts

**Figure 6:**
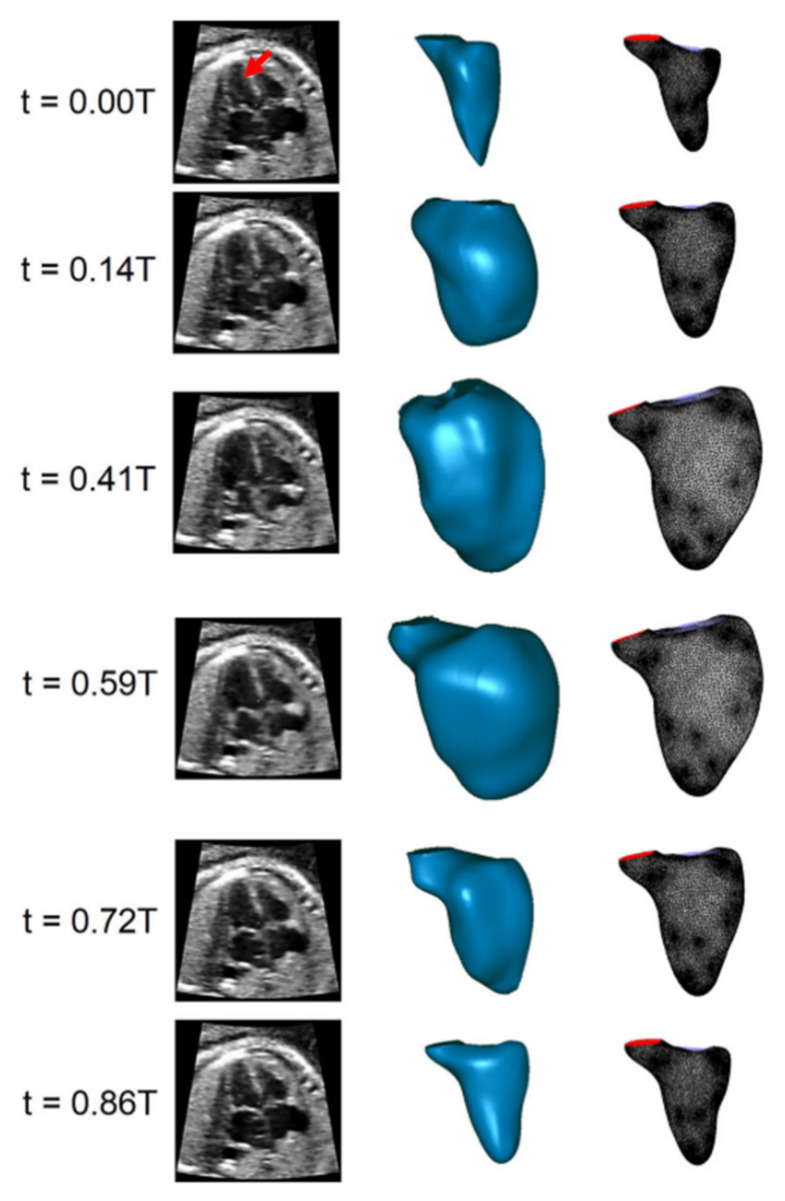
Ultrasound images of fetal hearts (**first column**). Reconstructed 3D models (**second column**) and generated meshes (**third column**) of fetal left ventricle over the cardiac cycle. Fetal left ventricle is shown by the red arrow in the ultrasound image at t = 0 T, where T is the period of one cardiac cycle (Reproduced from Lai et al. (2016) [41]).

Ventricle dynamics of human fetal hearts were investigated by a research group [35,40,41,47] using CFD models generated via 4D ultrasound scans. The movements of right and left ventricles are recorded during the cardiac cycle and applied as boundary conditions of dynamic mesh as shown in Figure 6 [41]. Prescribed wall motions on the ventricle squeeze the blood in the contraction phase. Since the wall positions change with time, a new mesh is generated for each time step.

**Figure 7:**
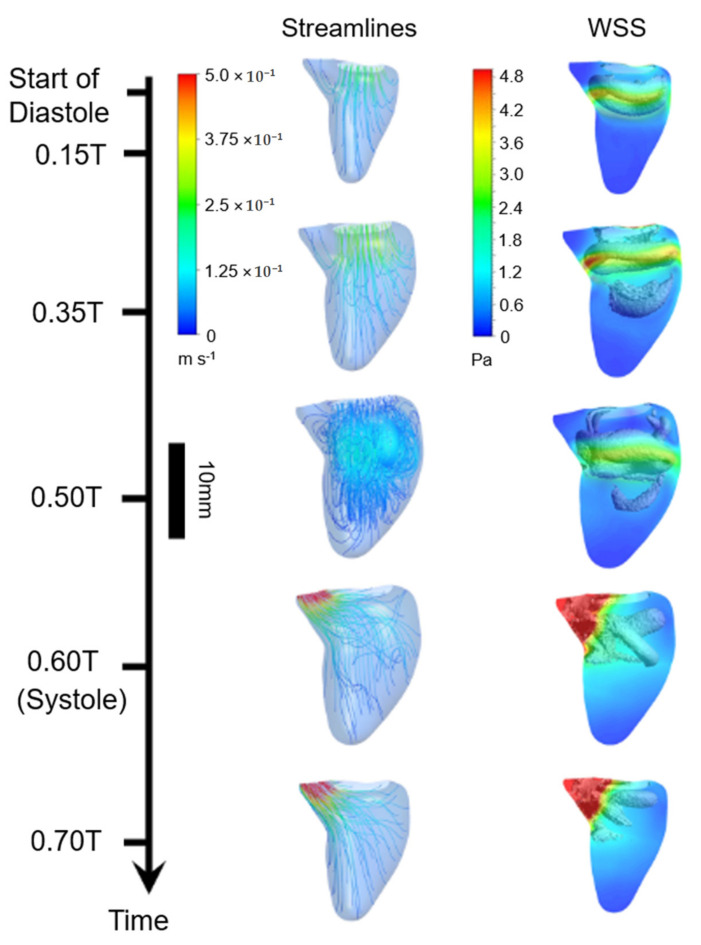
Flow streamlines and WSS patterns in a human fetal left ventricle considering various instants over the cardiac cycle. T is the period of cardiac cycle. In WSS plots, isosurfaces of vortex rings with recirculatory flow are shown. Relatively higher WSS levels are observed around the wall proximity of vortex rings (Reproduced from Lai et al. (2016) [41]).

Lai et al. (2016) investigated left ventricles of 20-week fetuses [41]. CFD results indicate that there are significant flow differences in adult and fetal hearts due to dissimilar morphology and heart rate. The heart rate of a healthy fetus is around 150 beats per minute; however, the normal adult heart rate is about 70 beats per minute, showing that the fetal heart rate is approximately twice of the adult heart rate [137]. In addition, the ratio between the systole and diastole periods is about 0.5 for the fetal left ventricle and about 0.7 for the adult left ventricle. Flow streamlines and WSS patterns in a fetal left ventricle are presented in Figure 7. Vortex-induced flow in ventricles increases the efficiency of diastolic filling and systolic pumping. The formation of vortices helps conserve the kinetic energy of the flow during the diastole. In adult ventricles, the energy of vortex structures is more dissipated compared to fetal ventricles due to higher cardiac cycle periods. Therefore, it can be stated that more flow energy is preserved in fetal heart for the subsequent cardiac cycle. For the studied three fetal hearts, average pressure difference between left ventricle and left atrium is found as 392 ± 344 Pa at peak diastole. The average pressure difference between the aorta and left ventricle is reported as 383 ± 314 Pa at the peak systole, and WSS is determined between 1 and 4 Pa in the fetal left ventricle [41].

Wiputra et al. (2016) investigated 20-week human right fetal ventricles using 4D CFD models [47]. Two main vortex structures were observed in the right ventricle and sustained until the systolic phase. It is stated that these prominent vortices conserve about 25% of peak diastolic kinetic energy for the subsequent cardiac cycle [35]. Due to the rotational flow in vortices, wall proximity of vortex flow is exposed to higher shear forces. According to the CFD results, WSS is within 0.4–1.2 Pa in the diastolic phase and 1.5–3.9 Pa in the systolic phase [47]. Peak WSS levels are observed on the regions close to the outflow tract. In a similar CFD study, diastolic and systolic WSS levels are reported around 1 Pa and within 2–4 Pa, respectively [40], which are in agreement with the previous findings. By averaging the results of three normal fetal right ventricles, peak pulmonary velocity is obtained as 0.380 ± 0.070 m/s [40]. In these CFD investigations, about 1 million tetrahedral elements are used in the dynamic meshes. For each cardiac cycle, 400 time steps with 0.001 s increments are employed, and a non-Newtonian fluid model is used for the blood using the Carreau–Yasuda model [35].

### 5.2. CFD Studies on Defected Human Fetal Hearts

Chen et al. (2017) modeled aortic coarctation of the human fetus [138]. Five normal fetal hearts at 32 weeks of gestation were used to simulate the hemodynamics in normal and constricted arches. The dimension of the aortic isthmus is digitally reduced by modifying the geometric model. It is observed that the progressive reduction in aortic isthmus resulted in an alteration in flow velocity, pressure, and WSS. When the aortic isthmus is reduced to 55% of its normal dimension, an exponential increase is observed in WSS and velocity. Therefore, 55% of reduction is considered as a threshold for hemodynamically significant aortic coarctation. The diameter of fetal aorta is greater than 1 mm, therefore a Newtonian fluid assumption can be used for the blood [138].

In a recent CFD study, Wiputra et al. (2018) modeled tetralogy of fallot (TOF) in a human fetal heart [35]. They performed flow simulations for 3 TOF (one 22-week and two 31-week hearts) and 7 normal fetal (four 22-week and three 31-week) hearts using ultrasound-based patient-specific model geometries. TOF is a combination of four defects which are pulmonary valve stenosis, ventricular septal defect, misplaced aorta, and increased right ventricular wall thickness. CFD results indicate that TOF increased diastolic WSS in right ventricles, on the other hand, no change is observed in left ventricle WSS levels. Two of three TOF hearts had an increased right ventricle wall thickness, but there was no change in left ventricle wall thicknesses between the normal and TOF hearts. It is reported that elevated pressure and WSS may have an influence on the thickening of the right ventricle wall. For the normal right ventricles, diastolic peak WSS is determined as 1.28 ± 0.61 Pa and 0.78 ± 0.14 Pa for 22-week and 31-week hearts, respectively, indicating a significant reduction in WSS with fetal heart development. In the opposite manner, diastolic peak WSS on the left ventricle increased from 0.74 ± 0.29 (at week 22) to 0.81 ± 0.19 (at week 31) [35]. These results demonstrate that WSS levels at right and left ventricles are significantly different at the early embryonic stage. However, as the fetus develops, a relatively uniform WSS distribution is observed in the left and right ventricles. The volumetric growth of the chambers is considered to be effective on uniform WSS distribution in the fetal heart. In Table 3, peak WSS levels predicted by CFD studies are presented for left and right ventricles of normal and TOF human fetal hearts considering different gestational stages.

## 6. Clinical Utility of CFD Simulations

Up to here, the altered hemodynamics and mechanobiological mechanisms in CHDs are investigated using CFD modeling. There are also clinical outcomes of simulated CFD models for determining possible treatments of CHDs [139]. Surgical planning of complex CHDs is one of the clinical outcomes of CFD modeling, where various operative techniques can be applied on a virtual environment to see the possible long-term effects of operations and to determine the optimum method by evaluating the post-operative hemodynamics [139]. As an example, Fontan repairs can be performed on a virtual environment using patient-specific anatomy, and CFD results of various trials can guide the clinicians to select the optimized operation to reduce pressure losses and improve the long-term outcome [139,140,141,142].

Another optimization approach using CFD modeling is the hemodynamic simulation of the Norwood procedure, which is performed for the treatment of HLHS [143]. There are no quantitative standards for the evaluation and prediction of the therapy and CFD simulations are performed before the surgery for improving the performance [144]. In addition to pre-surgical planning, CFD modeling enables personalized stent design in the treatment of pulmonary artery stenosis by utilizing patient-specific anatomy and virtual deployment techniques [145].

CFD simulations can also be used for non-invasive prediction of pressure gradient across the aortic coarctation site without the need for insertion of a catheter [146]. The aforementioned studies are conducted as case studies with few patients. Therefore, rigorous validation studies with large population models would better demonstrate the reliability of CFD modeling approaches [147].

With the advances in medical imaging modalities, data science, and machine learning algorithms, statistical shape models (SSM) can be generated to determine the anatomical mean shape and shape variations in CHDs which help classify the healthy and defected cases with increased accuracy [148]. CFD modeling can also be used in medical device development for CHD patients [147]. Growth models can be generated using CFD simulations in order to see the future effects of performed therapies [149,150]. These clinical utilities show the high potential of CFD modeling approaches in biomedical research related to CHDs.

## 7. Conclusions

In this paper, the main steps of CFD modeling during the embryonic cardiac development are explained and the findings of the recent CFD studies are summarized for chicken embryo, zebrafish embryo, and human fetus, as provided in Table 4. CHDs usually initiate during the initial stages of embryonic development and early fetal preventive interventions can potentially avoid further progress of the defects. In order to develop preventive actions, understanding the complete mechanism of CHD initiation and progression is a necessity. Genetic factors are considered as major causes of CHDs, but disturbed hemodynamics also plays a role in development of CHDs [151]. Altered hemodynamics changes the biomechanical environment and influence the growth and remodeling of cardiac cells since endocardial cells sense the shear stresses generated by the blood flow. The level of shear stress leads to up or down regulation of gene expressions which affect the heart morphology [152].

In order to understand the etiology of CHDs and monitor the embryonic heart development, animal models such as chicken and zebrafish are widely used. By performing micro-surgeries or using optical methods, cardiac defects are formed in embryos which resemble human CHDs. For in-depth analysis of parameters such as WSS, CFD models are employed using CT or MRI-based imaging of embryos. There are many CFD studies on chicken and zebrafish embryos, however, only a limited number of CFD studies are available on human fetal hearts due to the challenges in imaging. In recent studies, 4D CFD models are generated using the ultrasound-based images taken over the entire cardiac cycle. By using the prescribed wall motion of the heart wall, dynamic meshes are used to determine accurate solutions. The findings of these studies confirm the influence of mechanical parameters, particularly WSS, on the fetal cardiac development. In the current human fetal heart models, solid mechanics of heart tissues are not considered and the governing flow equations are solved using dynamic CFD meshes. In future studies, incorporating the FSI approach into fetal heart models can provide broader perspectives on CHDs.

## Figures and Tables

**Table 1:** Wall shear stress (WSS) levels in normal chicken embryos for various developmental stages. Values in parentheses are standard deviations (OFT: outflow tract, AV canal: atrioventricular canal, RVOFT: right ventricle outflow tract, LVOFT: left ventricle outflow tract).

Embryonic Stage–Investigated Region	Peak WSS (Dynes/cm^2^)	Spatially Averaged WSS at Peak Velocity (Dynes/cm^2^)	Spatially and Temporally Averaged WSS (Dynes/cm^2^)
HH16–OFT [32]	18.16 (3.18)	9.55 (0.40)	3.03 (0.11)
HH17–AV canal [95]	19.34 (4.45)	9.17 (3.2)	3.62 (0.32)
HH18–OFT [104]	60		
HH18–Aortic Arch II left side [103]	-	59.4 (14)	17.9 (5)
HH18–Aortic Arch II right side [103]	-	47.1 (12)	14.3 (4)
HH18–Aortic Arch III left side [103]	-	64.4 (18)	19.4 (5)
HH18–Aortic Arch III right side [103]	-	55.8 (17)	17.0 (5)
HH18–Aortic Arch IV left side [103]	-	41.7 (17)	12.5 (5)
HH18–Aortic Arch IV right side [103]	-	59.9 (18)	18.2 (6)
HH21–OFT [96]	31	-	-
HH23–AV canal [95]	78.33 (37.09)	33.59 (16.84)	6.79 (3.22)
HH23–Proximal OFT [32]	59.36 (10.07)	28.15 (7.47)	4.23 (0.09)
HH23–Distal OFT [32]	57.12 (9.04)	31.69 (7.48)	7.05 (0.82)
HH24–Aortic Arch III left side [103]	-	104.9 (37)	23.6 (8)
HH24–Aortic Arch III right side [103]	-	82.2 (16)	18.4 (4)
HH24–Aortic Arch IV left side [103]	-	92.3 (56)	20.8 (12)
HH24–Aortic Arch IV right side [103]	-	122.9 (40)	27.5 (9)
HH24–Aortic Arch VI left side [103]	-	130.7 (28)	29.3 (6)
HH24–Aortic Arch VI right side [103]	-	40.4 (40)	9.0 (9)
HH27–AV canal [95]	250.09 (51.49)	59.7 (4.6)	6.1 (0.52)
HH27–OFT [32]	236.07 (39.34)	111.74 (21.7)	39.49 (9.34)
HH27–Carotid arteries [92]	-	4.60	-
HH27–Pharyngeal aortic arches [92]	-	18.90	-
HH27–Left and right dorsal aortae [92]	-	2.4	-
HH27–Common dorsal aorta [92]	-	7.3	-
HH30–AV canal [95]	287.18 (67.45)	86.27 (8.6)	9.11 (1.061)
HH30–RVOFT [32]	671.24 (211.36)	184.36 (34.26)	100.67 (27.82)
HH30–LVOFT [32]	400.93 (65.65)	226.67 (20.41)	136.5 (17.82)

**Table 2:** WSS levels in normal zebrafish embryos for various developmental stages (AV canal: atrioventricular canal).

Embryonic Stage–Investigated Region	Peak WSS (Dynes/cm^2^)
20–30 hpf–AV canal [133]	3.5
48 hpf–AV canal [126]	70
40–50 hpf–AV canal [133]	20
52 hpf–Ventral vein [39]	3.4
60–70 hpf–AV canal [133]	28
80–90 hpf–AV canal [133]	58
108 hpf–AV canal [134]	13.6
110–120 hpf–AV canal [133]	82
120 hpf–Mid-ventricular segment [132]	4–11
120 hpf–Ventricle inflow tract [132]	130
120 hpf–Ventricle outflow tract [132]	110

**Table 3:** Comparison of peak WSS levels of normal and three TOF (tetralogy of fallot) human fetal hearts at different gestational stages (LV: left ventricle, RV: right ventricle).

Gestation Week–Heart Condition	Diastolic RV Peak WSS (Dynes/cm^2^)	Diastolic LV Peak WSS (Dynes/cm^2^)	Systolic RV Peak WSS (Dynes/cm^2^)	Systolic LV Peak WSS (Dynes/cm^2^)
22 week–TOF-I [35]	27.0	9.3	11.6	11.2
31 week–TOF-II [35]	12.6	9.4	15.8	15.1
31 week–TOF-III [35]	12.8	5.6	16.6	13.9
22 week–Normal [35]	12.8 (6.1)	7.4 (2.9)	20.0 (2.8)	15.1 (2.6)
31 week–Normal [35]	7.8 (1.4)	8.1 (1.9)	16.1 (1.8)	15.8 (3.8)
20 week–Normal [47]	12.0	-	39.0	-

**Table 4:** Summary of CFD studies presented in this review.

	Model Type	Embryonic Development Stage	Health Status of Investigated Embryonic Hearts
Bharadwaj et al. (2012) [32]	Chicken embryo	HH16, HH23, HH27, HH30	Normal
Yalcin et al. (2011) [95]	Chicken embryo	HH17, HH23, HH27, HH30	Normal
Wang et al. (2009) [103]	Chicken embryo	HH18, HH24	Normal
Liu et al. (2012) [104]	Chicken embryo	HH18	Normal
Liu et al. (2007) [96]	Chicken embryo	HH21	Normal
Tan et al. (2015) [92]	Chicken embryo	HH27	Normal
Ho et al. (2017) [105]	Chicken embryo	HH25	Normal
Kowalski et al. (2014) [106]	Chicken embryo	HH21	Normal, Left atrial ligated
Lindsay et al. (2015) [107]	Chicken embryo	HH18, HH24	Normal, Occluded pharyngeal arch artery
Menon et al. (2015) [111]	Chicken embryo	HH16/17	Normal, Constricted ventricle junction/outflow tract
Lee et al. (2013) [133]	Zebrafish embryo	20–30 hpf, 40–50 hpf, 60–70 hpf, 80–90 hpf, 110–120 hpf	Normal
Boselli and Vermot (2016) [126]	Zebrafish embryo	48 hpf	Normal
Gomez-Garcia et al. (2018) [39]	Zebrafish embryo	52 hpf	Normal
Miller (2011) [134]	Zebrafish embryo	108 hpf	Normal
Foo et al. (2020) [132]	Zebrafish embryo	120 hpf	Normal
Vedula et al. (2017) [31]	Zebrafish embryo	4 dpf	Normal, Inhibited trabeculaction, Inhibited proliferation, Inhibited ventricle development
Lee et al. (2018) [15]	Zebrafish embryo	4 dpf	Normal, Inhibited trabeculation, Inhibited contractility
Wiputra et al. (2018) [35]	Human fetus	22 week, 31 week	Normal, Tetralogy of fallot
Wiputra et al. (2016) [47]	Human fetus	20 week	Normal
Lai et al. (2016) [41]	Human fetus	20 week	Normal
Wiputra et al. (2016) [40]	Human fetus	20 week	Normal
Chen et al. (2017) [138]	Human fetus	32 week	Normal, Aortic coarctation
